# Subcutaneous immunoglobulin-induced eczematous dermatitis

**DOI:** 10.1016/j.jdcr.2024.12.006

**Published:** 2024-12-16

**Authors:** Rachel Breidbart, Matthew D. Vesely

**Affiliations:** Department of Dermatology, Yale School of Medicine, New Haven, Connecticut

**Keywords:** dermatitis, drug reactions, eczema, SCIG, subcutaneous immunoglobulin

## Introduction

Human polyclonal immunoglobulin therapy has been a long-standing, effectual treatment for immunodeficiency and autoimmune diseases. The 2 most frequent routes for administering immunoglobulin are intravenous immunoglobulin (IVIG) and subcutaneous immunoglobulin (SCIG).^1^ Both SCIG and IVIG are generally well tolerated, however SCIG has been shown to cause fewer systemic adverse effects.^2^ The most common side effect of SCIG is brief swelling at the site of infusion, whereas other rare complications include thromboembolic events, hemolysis, aseptic meningitis, and transfusion associated acute lung injury.^3^ Uncommonly, adverse dermatologic reactions to IVIG administration have been noted in the literature. These rashes are typically urticarial, morbilliform, and desquamated,^4^ although rare eczematous rashes have also been reported.^3^, ^4^, ^5^, ^6^, ^7^ In contrast, there have been much fewer reported cases of adverse dermatologic reactions to SCIG outside of transient, local, inflammatory responses.^2^ Here, we report a case of an eczematous eruption on the palms and soles that developed after SCIG administrations that is reminiscent of IVIG-induced eczema.

## Case report

**Fig 1:**
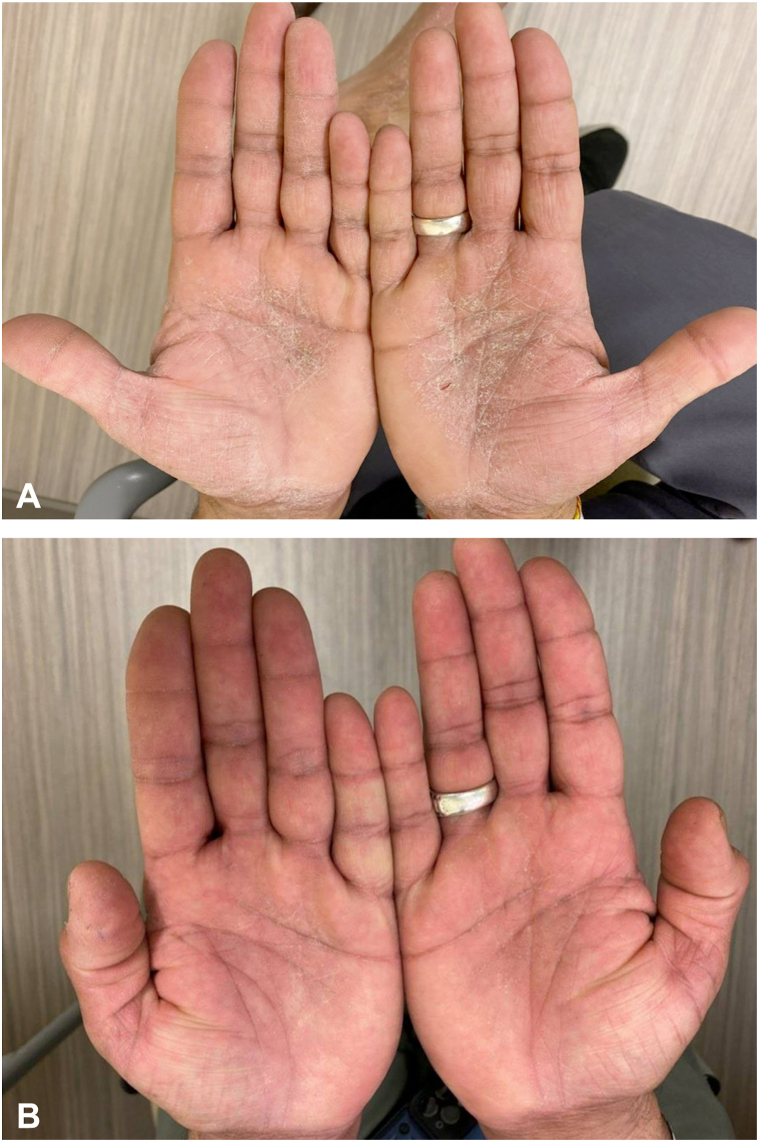
Clinical images. Lesions on hands (**A**) before and (**B**) 2 weeks after subcutaneous immunoglobulin (SCIG) formulations were switched from SCIG 20% Hizentra to 16.5% Cutaquig for the treatment of SCIG-induced eczematous dermatitis. Patient also using ruxolitinib 1.5% cream.

**Fig 2:**
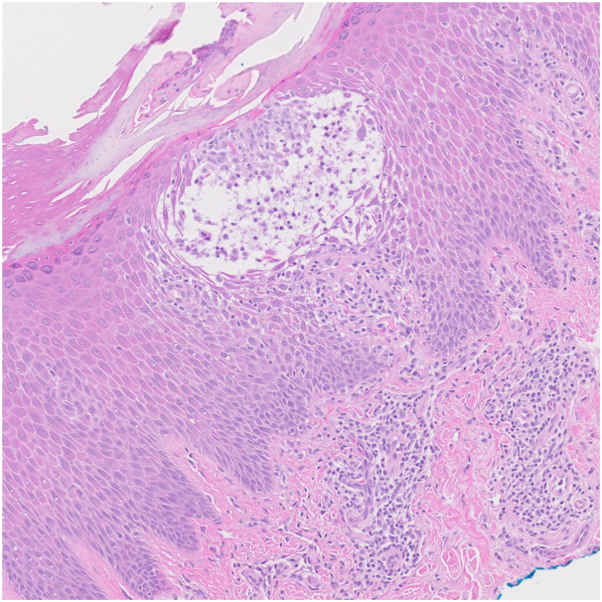
Skin biopsy specimen. Spongiosis and inflammatory infiltrates were found on histologic analysis. (Hematoxylin-eosin stain; original magnification: ×100.)

A 59-year-old man with combined variable immunodeficiency, autoimmune hemolytic anemia with subsequent splenectomy, diabetes mellitus, hyperlipidemia, hypertension, and hypogammaglobulinemia was placed on SCIG 20% (Hizentra) 0.4 g/kg every 2 weeks. He was diagnosed with combined variable immunodeficiency 2 months before starting SCIG and received a single dose of IVIG 8 months prior for autoimmune hemolytic anemia. Two months after starting SCIG infusions, the patient experienced a pruritic, scaly rash on his hands and feet. A flare of the rash would occur 1 to 2 days after each SCIG treatment. He had no history of hand dermatitis, and lacked other exposures that would account for his eczematous reaction. Physical examination revealed confluent pink, scaly, lichenified plaques involving the palms and soles (Fig 1, *A*). A shave biopsy from right palm revealed acanthosis, spongiosis with vesiculation, parakeratosis, and an inflammatory infiltrate with lymphocytes predominantly compatible with an eczematous process (Fig 2). Other differential diagnoses such as tinea, and scabies, was considered less likely because of lack of burrows on examination. Furthermore, histologic examination did not show dermatophytes or mites, and a periodic acid–Schiff stain was negative for fungal elements. Contact dermatitis may be considered as a potential cause for his rash, no new contactants were identified based on history.

He was first treated with clobetasol 0.05% ointment for weeks with little improvement. Next, he used topical ruxolitinib 1.5% cream for several weeks, which led to mild improvement in the itch and rash, but the eczema persisted. At this time, his SCIG formulation was switched from SCIG 20% Hizentra to 16.5% Cutaquig, which helped to reduce the extent of his eczematous dermatitis (Fig 1, *B*). He has been on Cutaquig for 18 months with very mild and intermittent palmar eczematous dermatitis that he treats with ruxolitinib 1.5% cream as needed.

## Discussion

Although eczematous reactions to IVIG treatment are progressively being identified, there has been a delay in reports for SCIG. This may be because IVIG administration has long been the prevailing route for immunoglobulin replacement, but only in the last 3 decades has SCIG become more regularly used.^8^ Another reason for this disparity in recognized cases may be differences in pharmaceutical properties of the products, pharmacokinetics of immunoglobulin, and frequency of infusions between the 2 routes of administration. With fewer treatments, IVIG produces higher peak concentrations and lower trough concentrations, whereas more frequent SCIG infusions result in steady immunoglobulin plasma concentrations, subsequently leading to fewer systemic adverse effects.^9^ As SCIG becomes increasingly used to replace IVIG, additional cases of eczematous dermatitis may be reported.

A speculative mechanism by which this eczematous reaction develops begins with increased plasma levels of interleukin (IL) 33, IL-4, and IL-13, which immunoglobulin therapy has been shown to generate. This may be attributed to activation of T helper 2 cells, B cells, and dendritic cells. IL-33 can further induce IL-13 production by T helper 2 cells, adding to the circulating plasma levels of IL-4 and IL-13.^10^ Elevations in both cytokines constitute a hallmark of atopic dermatitis and may contribute to the patient’s presentation with eczematous dermatitis.

Similar to reported cases of IVIG-induced eczema, subsequent SCIG administration resulted in greater severity of the rash.^4^^,^^5^^,^^7^ Local pompholyx reactions on the palms are the most prevalently recognized eczematous adverse reactions to immunoglobulin in the literature.^4^, ^5^, ^6^ In this report, we describe a case of a localized eczematous eruption with pompholyx on the hands and feet as a result of SCIG treatment. Our patient did not have a prior history of atopic disorders, which is a consistent feature of patients who experience immunoglobulin-induced eczematous reactions.^4^^,^^6^

Topical steroids are typically used to treat mild cases of immunoglobulin-induced eczema; nevertheless, in some severe cases, immunoglobulin therapy is discontinued.^4^, ^5^, ^6^, ^7^ Studies have also found mixed results regarding the benefits of switching immunoglobulin preparations to prevent recurrence.^7^^,^^8^ Our patient responded poorly to topical steroids and topical Janus kinase inhibitor, ruxolitinib 1.5% cream while on Hizentra SCIG. There was improvement with switching to a lower concentrated preparation of immunoglobulin, from 20% Hizentra to 16.5% Cutaquig, supporting the benefit of decreasing SCIG concentrations to avoid repeated eczematous eruptions.

This case demonstrates that eczematous rashes are potential adverse reactions to SCIG treatment and are not solely IVIG-induced. Such reactions may improve by changing SCIG preparations to lower concentrations, allowing the rash to subside without discontinuing treatment. As SCIG use increases in popularity, more awareness, and recognition of eczematous reactions as an adverse effect of SCIG are needed.

## Conflicts of interest

Dr Vesely’s spouse is an employee of Regeneron Pharmaceuticals. Author Breidbart has no conflicts of interest to declare.
